# Evolution and Clinical Impact of Prosthodontic Appliances: A Narrative Review of Splints, Stents, and Oral Appliances

**DOI:** 10.7759/cureus.113602

**Published:** 2026-07-29

**Authors:** Sayali P Belkhode, Prashanth Shetty, Ayushi R Gupta, Anisha Nayak, Anmol Tiwari

**Affiliations:** 1 Prosthodontics, Triveni Institute of Dental Sciences Hospital and Research Center, Bilaspur, IND

**Keywords:** cad/cam, digital dentistry, occlusal splints, oral appliances, prosthodontic rehabilitation, surgical stents

## Abstract

Splints, stents, and oral appliances play an important supportive role in modern prosthodontic practice, contributing to diagnosis, treatment stabilization, surgical accuracy, and functional rehabilitation. Over time, these appliances have evolved from largely empirical devices into precision-based therapeutic tools guided by biomechanical principles and digital technologies. Recent developments in computer-aided design and manufacturing (CAD/CAM), three-dimensional printing, and evidence-based appliance therapy have improved their accuracy, consistency, and clinical predictability.

Occlusal splints continue to be widely used as conservative, reversible treatment options for temporomandibular disorders and bruxism. Digital fabrication techniques have demonstrated improved fit and reproducibility when compared with conventional fabrication methods. Surgical and radiotherapeutic stents have expanded the role of prosthodontists in interdisciplinary implant and oncologic rehabilitation by improving procedural precision and protecting surrounding tissues. In addition, oral appliance therapy has gained increasing acceptance in the management of obstructive sleep apnea, particularly in patients who are intolerant of continuous positive airway pressure therapy.

Despite their widespread use, considerable variation remains in appliance design, material selection, and clinical protocols, which may influence treatment outcomes. Recent literature highlights the importance of individualized appliance therapy, digital workflow integration, and a deeper understanding of neuromuscular and biomechanical mechanisms.

This narrative review summarizes the current evidence on the evolution, mechanisms of action, clinical applications, and technological advancements of prosthodontic appliances, with particular emphasis on splints, stents, oral appliance therapy, and digital technologies. It aims to provide clinicians and postgraduate students with a comprehensive overview of the expanding role of these appliances in contemporary prosthodontic practice.

## Introduction and background

Prosthodontic appliances, including splints, stents, and oral appliances, form an integral component of contemporary prosthodontic practice. They are widely used for diagnosis, therapeutic stabilization, surgical guidance, tissue protection, and functional rehabilitation. Depending on their clinical application, these appliances modulate neuromuscular activity, optimize occlusal force distribution, improve airway patency, facilitate surgical precision, and support tissue healing. Appropriate selection and design are therefore essential for achieving predictable outcomes in the management of temporomandibular disorders (TMDs), implant rehabilitation, maxillofacial defects, and sleep-related breathing disorders [1-3].

Occlusal splints are commonly used as conservative, reversible treatment options for TMDs and bruxism. By reducing muscle hyperactivity and protecting the dentition, they help alleviate pain and prevent further structural damage [4,5]. Surgical and radiotherapeutic stents, on the other hand, improve procedural accuracy while minimizing injury to adjacent tissues, thereby enhancing postoperative recovery and overall quality of life [6,7]. Oral appliances, particularly mandibular advancement devices, have also emerged as effective treatment alternatives for obstructive sleep apnea, offering improved patient acceptance in selected cases [8-10]. The successful clinical performance of prosthodontic appliances depends on appropriate case selection, accurate design, and meticulous clinical application [11].

Historically, prosthodontic appliances were developed largely through empirical methods, with fabrication techniques based on clinical experience rather than scientific validation. Early occlusal splints, often constructed from metal wires and basic acrylic materials, were primarily intended to stabilize teeth and protect occlusal surfaces [12]. Similarly, early surgical stents served as approximate mechanical guides with limited accuracy [13]. Oral appliances initially evolved from orthodontic functional devices, with their role in airway management being recognized only later [14].

With advances in prosthodontics in the mid-20th century, appliance design gradually incorporated principles of biomechanics, neuromuscular physiology, and functional occlusion. Occlusal splints began to be used not only for protection but also for muscle relaxation and joint stabilization [4,15]. Surgical stents became more refined, particularly with the introduction of implant dentistry, allowing prosthetic planning to guide surgical placement [13]. At the same time, functional oral appliances were increasingly adapted for the management of sleep-disordered breathing [8].

The late 20th and early 21st centuries marked a significant shift toward evidence-based practice and digital integration. The introduction of cone-beam computed tomography (CBCT), intraoral scanning, computer-aided design and manufacturing (CAD/CAM) systems, and three-dimensional printing transformed appliance fabrication by enabling patient-specific designs with improved accuracy and reproducibility [16-18]. More recently, artificial intelligence-assisted planning and advanced biomaterials have further expanded the scope of prosthodontic appliance therapy [19,20].

Despite these advancements, considerable variability persists in appliance design protocols, fabrication methods, and clinical applications. Conventional and digital techniques often coexist without standardized guidelines, and comparative long-term evidence remains limited [20]. Although numerous reviews have examined specific categories of prosthodontic appliances, integrated narrative reviews encompassing their evolution, clinical applications, and contemporary digital advancements remain limited. Therefore, this narrative review aims to provide a comprehensive overview of the evolution, classification, mechanisms of action, clinical applications, and technological advances in prosthodontic appliances; critically assess current limitations; and outline future directions in prosthodontic appliance therapy.

Review methodology

This manuscript is a narrative review of the literature. A literature search was conducted using electronic databases, including PubMed, Scopus, Web of Science, and Google Scholar. Relevant English-language articles, reviews, clinical studies, textbooks, and professional guidelines published over the past several decades were considered. The search included keywords such as “prosthodontic appliances,” “occlusal splints,” “stents,” “oral appliance therapy,” “implant surgical guides,” “CAD/CAM,” “3D printing,” and “digital dentistry.” Studies relevant to the evolution, classification, clinical applications, and technological advances in prosthodontic appliances were included to provide a comprehensive overview of the subject.

## Review

Prosthodontic appliances encompass a broad spectrum of devices designed to modify occlusal relationships, guide surgical procedures, protect oral tissues, and facilitate functional rehabilitation. Based on their therapeutic objectives, design characteristics, and clinical applications, these appliances can be systematically classified as shown in Table 1.

**Table 1:** Proposed clinical classification of prosthodontic appliances. TMD: temporomandibular disorders, CAD: computer-aided design, CAM: computer-aided manufacturing.

Category	Examples	Primary Clinical Application
Occlusal Splints	Stabilization splint, anterior bite plane, repositioning splint	TMD, bruxism, occlusal stabilization
Surgical & Implant Stents	Implant guide, radiotherapy stent, surgical guide	Surgical guidance, tissue protection
Maxillofacial Prosthodontic Appliances	Palatal lift, speech bulb, feeding plate	Rehabilitation of acquired or congenital defects
Oral Appliance Therapy	Mandibular advancement device, tongue-retaining device	Obstructive sleep apnea, snoring
Digital Prosthodontic Appliances	CAD/CAM surgical guides, 3D-printed splints	Digitally guided rehabilitation

The proposed clinical classification provides clinicians with a practical framework for understanding the intended function of each appliance group and facilitates appropriate appliance selection based on specific clinical requirements. Although some categories may overlap in function, this systematic framework improves clarity in diagnosis, treatment planning, interdisciplinary communication, and clinical decision-making.

Occlusal splints: classification, mechanisms, and clinical applications

Occlusal splints are widely used prosthodontic appliances that serve both diagnostic and therapeutic purposes in the management of TMDs, bruxism, and occlusal discrepancies. These appliances function by reducing abnormal muscle activity, redistributing occlusal forces, and protecting the dentition and associated structures from parafunctional damage [1,2].

According to established prosthodontic classifications, occlusal splints can be categorized as stabilization appliances, anterior positioning appliances, anterior bite planes, posterior bite planes, and pivoting appliances [14]. These classifications correspond to their functional roles in modifying mandibular position, occlusal relationships, and temporomandibular joint loading.

Stabilization splints (Michigan splints) are the most commonly used appliances and are typically fabricated for the maxillary arch. They provide uniform and simultaneous occlusal contacts when the condyles are in a musculoskeletally stable position. Their primary objective is to eliminate orthopedic instability between the occlusion and joint position, thereby reducing muscle hyperactivity and associated pain [1,2,16].

Anterior repositioning splints are indicated in cases of internal derangement, particularly disc displacement with reduction. These appliances position the mandible anteriorly, improving the condyle-disc relationship and facilitating adaptive remodeling of the retrodiscal tissues. However, prolonged use may result in occlusal changes and therefore requires careful monitoring [14].

Anterior bite planes are designed to contact only the anterior teeth, thereby disengaging the posterior occlusion and reducing elevator muscle activity. These appliances are useful in managing acute occlusal disturbances and muscle disorders; however, long-term use may lead to posterior supraeruption and anterior open bite [14].

Posterior bite planes are indicated in cases requiring alteration of the vertical dimension or mandibular repositioning. They provide occlusal contact in the posterior region and may be useful in selected cases of disc derangement; however, their use is limited by potential complications such as occlusal instability [14].

Pivoting appliances are designed with a single posterior point of contact, theoretically reducing intra-articular pressure within the temporomandibular joint. Despite their proposed biomechanical advantages, clinical evidence supporting their routine use remains limited [14].

Soft or resilient splints are fabricated from flexible materials and are primarily used for short-term protection in patients with bruxism or those at risk of trauma. Although they improve comfort, some studies suggest that they may increase muscle activity; therefore, their use should be carefully considered [16].

Nociceptive trigeminal inhibition (NTI) splints are anterior bite devices that prevent posterior tooth contact and reduce elevator muscle activity. These appliances have been used in the management of bruxism and certain types of orofacial pain, including tension-type headaches [17].

Specialized splints in prosthodontics

Beyond conventional occlusal splints, several specialized splints are used in prosthodontics for specific therapeutic, surgical, and rehabilitative purposes. Unlike occlusal splints, which primarily manage TMDs and parafunctional habits, specialized splints are indicated for the stabilization of teeth, management of maxillofacial trauma, and guidance during surgical procedures. Their design is dictated by the underlying pathology, treatment objectives, and anticipated functional requirements.

Periodontal splints are indicated for teeth exhibiting increased mobility secondary to periodontal attachment loss. By connecting adjacent teeth into a single functional unit, these appliances distribute occlusal forces more evenly, reduce mobility, improve patient comfort, and facilitate periodontal healing when combined with appropriate periodontal therapy. Contemporary periodontal splints may be fabricated using fiber-reinforced composite materials, orthodontic wire-composite systems, or cast metal frameworks, depending on the severity of mobility and long-term treatment requirements.

Dentoalveolar fracture splints provide temporary immobilization of fractured teeth and alveolar bone following traumatic injuries. Their primary objective is to stabilize fracture segments while preserving the existing occlusal relationship and allowing physiological bone healing. Commonly used designs include arch-bar splints, cast cap splints, acrylic splints, and wire-composite splints. The choice of splint depends on the fracture configuration, dentition status, and need for simultaneous maxillomandibular fixation.

Occlusal wafer splints are routinely used during orthognathic and reconstructive maxillofacial surgery. Fabricated using preoperative diagnostic casts or digital surgical planning, these appliances accurately transfer the planned maxillomandibular relationship to the operative field. They facilitate intraoperative jaw positioning, intermaxillary fixation, and postoperative occlusal stabilization, thereby improving surgical precision and minimizing postoperative occlusal discrepancies.

Gunning splints remain a preferred treatment modality for edentulous patients presenting with mandibular or maxillary fractures. These appliances immobilize fractured jaw segments while maintaining the predetermined maxillomandibular relationship. Depending on the clinical situation, one-piece, two-piece, sectional, or modified Gunning splints may be fabricated to permit nutrition, maintenance of oral hygiene, and functional rehabilitation during fracture healing.

Stents in prosthodontics

Stents are specialized prosthodontic appliances designed to guide surgical procedures, protect oral tissues, deliver therapeutic agents, or maintain anatomical structures during healing. They represent an important extension of prosthodontic practice into implant dentistry, maxillofacial rehabilitation, and head and neck oncology by translating prosthetic planning into precise clinical execution. Over the past two decades, stent fabrication has evolved from manually fabricated acrylic devices to digitally designed and CAD/CAM-manufactured appliances, resulting in substantial improvements in surgical precision, reproducibility, and patient outcomes.

Based on their clinical applications, prosthodontic stents may be broadly classified as implant surgical stents, radiotherapy stents, fluoride carrier stents, periodontal stents, antihemorrhagic stents, drainage stents, nasal stents, trismus stents, and tissue-conditioning stents. Each category serves a distinct therapeutic purpose while contributing to improved treatment predictability and preservation of the surrounding tissues.

Implant surgical guides

Implant surgical guides constitute one of the most significant advances in contemporary implant prosthodontics by enabling prosthetically driven implant placement with high precision and predictability. Their primary objective is to accurately transfer the virtual implant treatment plan to the clinical setting while maintaining the planned implant position, angulation, depth, and restorative orientation. Compared with conventional freehand surgery, guided implant placement improves prosthetic outcomes, reduces surgical complications, and enhances long-term treatment success [18].

Traditionally, surgical guides were fabricated manually using diagnostic casts, vacuum-formed templates, and autopolymerizing acrylic resin. Although these guides provided basic orientation during implant placement, they were associated with limited accuracy and operator-dependent variability. The integration of CBCT, intraoral scanning, virtual implant planning software, CAD/CAM technology, and additive manufacturing has transformed implant guide fabrication into a highly precise digital workflow.

**Figure 1:**
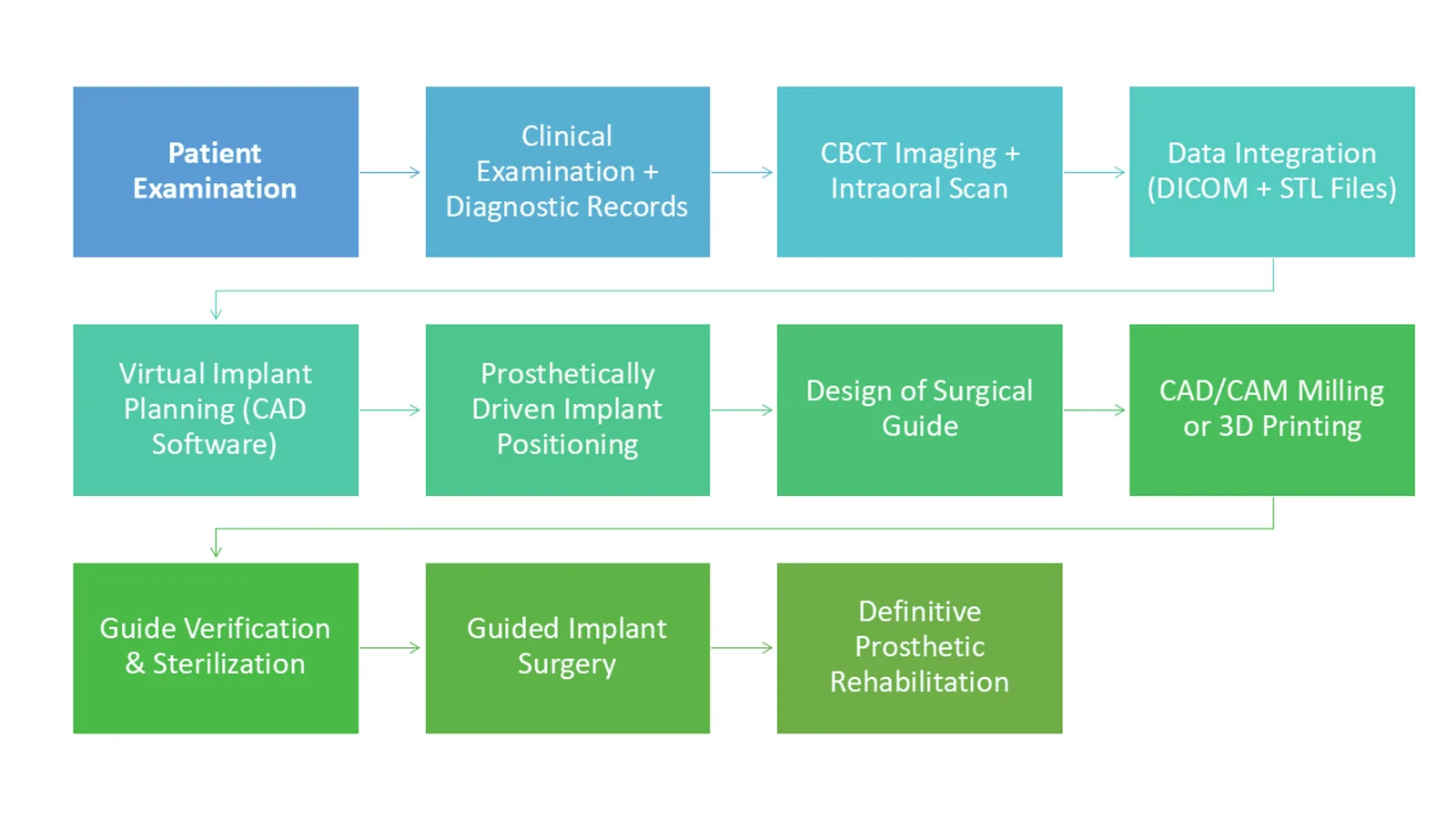
Digital workflow for fabrication and clinical application of implant surgical guides. CBCT: cone-beam computed tomography, DICOM: Digital Imaging and Communications in Medicine, STL: Standard Tessellation Language, CAD/CAM: computer-aided design/computer-aided manufacturing.

A contemporary digital workflow begins with the acquisition of a CBCT scan and an intraoral digital scan or digitized master cast (Figure 1). These datasets are merged using dedicated implant planning software, allowing prosthetically driven virtual implant positioning while simultaneously evaluating bone availability, anatomical limitations, restorative space, and occlusal requirements. The finalized treatment plan is subsequently converted into a three-dimensional surgical guide using CAD/CAM milling or, more commonly, three-dimensional printing.

Implant surgical guides are broadly classified according to the extent of surgical guidance they provide: Pilot-drill guides direct only the initial osteotomy and require subsequent drilling to be performed freehand. Partially guided systems control pilot drilling and selected sequential drills while allowing flexibility during implant insertion. Fully guided systems provide guidance throughout the entire osteotomy preparation and implant placement, ensuring maximal positional accuracy.

Dynamic navigation systems use real-time computer-assisted tracking to guide implant placement without a physical surgical template, allowing intraoperative adjustments while maintaining high accuracy [19,20].

Several commercially available planning platforms have significantly improved virtual treatment planning by integrating CBCT imaging with digital impressions and restorative designs. These systems facilitate prosthetically driven implant placement, virtual wax-ups, assessment of restorative emergence profiles, and simulation of implant-supported prostheses before surgery.

Clinical studies consistently demonstrate that guided implant surgery reduces angular deviation, coronal and apical positional deviations, and depth discrepancies compared with conventional freehand placement. Guided surgery also permits minimally invasive flapless implant placement in carefully selected cases, resulting in reduced surgical time, decreased postoperative discomfort, preservation of the soft tissue architecture, and faster patient recovery. Improved precision further contributes to optimal prosthetic emergence profiles, enhanced esthetics, and increased long-term implant success [18-20].

Despite these advantages, guided implant surgery is not without limitations. Cumulative errors arising from CBCT image acquisition, optical scanning, data merging, software planning, guide fabrication, and sleeve tolerance may influence the final implant position. Restricted mouth opening, inadequate interarch space, metallic artifacts, and increased treatment costs may also limit its clinical application. Consequently, meticulous case selection, accurate digital planning, verification of guide stability, and clinician experience remain essential for achieving predictable outcomes [19,20]. 

The workflow illustrates the contemporary digital protocol, beginning with clinical examination, CBCT imaging, and intraoral scanning, followed by data integration, prosthetically driven virtual implant planning, CAD/CAM-based guide fabrication, guided implant surgery, and definitive prosthetic rehabilitation [18-20].

Radiotherapy and therapeutic stents

Radiotherapy and therapeutic stents are indispensable adjuncts in the prosthodontic management of patients undergoing treatment for head and neck malignancies. These appliances are designed to improve the precision of radiation delivery, protect healthy oral tissues from unnecessary irradiation, and enhance patient comfort throughout treatment. Prosthodontists play a vital role in the fabrication of these customized devices through close collaboration with radiation oncologists and maxillofacial surgeons.

Radiotherapy stents are primarily used to reposition or displace normal oral structures away from the radiation field, thereby minimizing radiation-induced damage to healthy tissues. Tissue-displacing stents physically separate radiosensitive structures such as the tongue, palate, lips, cheeks, salivary glands, and mandible from the treatment area. This significantly reduces the incidence and severity of radiation-induced complications, including oral mucositis, xerostomia, dysgeusia, trismus, and osteoradionecrosis, while improving treatment reproducibility and radiation accuracy [9,10].

Positioning stents ensure the reproducible positioning of the jaws and oral soft tissues during repeated radiotherapy sessions. By maintaining a consistent spatial relationship between the patient and the radiation beam, these appliances improve treatment precision while minimizing geometric errors throughout the course of radiotherapy.

Fluoride carrier stents are fabricated using vacuum-formed thermoplastic materials and serve as reservoirs for topical fluoride gels. The daily application of neutral sodium fluoride using these carriers significantly reduces the incidence of radiation-induced dental caries, enamel demineralization, and root surface destruction in patients receiving radiotherapy for head and neck cancer [9].

Recent advances in digital dentistry have further enhanced radiotherapy stent fabrication through CAD/CAM technology and three-dimensional printing. Digital workflows enable improved adaptation, greater reproducibility, shorter laboratory time, and enhanced communication among prosthodontists, radiation oncologists, and maxillofacial surgeons, thereby improving overall treatment quality [21].

Other clinical applications of prosthodontic stents

Beyond implant surgery and radiotherapy, prosthodontic stents have numerous applications in oral surgery, periodontology, maxillofacial rehabilitation, and postoperative care. Their primary purposes are to protect healing tissues, maintain anatomical form, improve surgical outcomes, and facilitate functional rehabilitation.

Antihemorrhagic stents are fabricated following extensive oral surgical procedures to provide controlled pressure over the surgical site, thereby reducing postoperative bleeding and stabilizing blood clot formation. These appliances are particularly valuable in patients receiving anticoagulant therapy or those with inherited bleeding disorders [10].

Periodontal stents are commonly used following periodontal surgery to stabilize periodontal dressings, protect surgical sites from mechanical trauma, and support uneventful wound healing. They also improve patient comfort by minimizing irritation during mastication and speech.

Drainage stents facilitate the continuous evacuation of exudates from cystic cavities, postoperative dead spaces, or infected surgical sites. By preventing the premature closure of drainage pathways, these appliances reduce the risk of recurrent infection and promote progressive healing.

Compression stents provide continuous controlled pressure over soft tissues following reconstructive procedures or skin grafting or during the management of hypertrophic scars and keloids. Sustained compression minimizes edema, improves tissue adaptation, and enhances esthetic outcomes.

Nasal stents are used following rhinectomy or nasal reconstructive procedures to maintain airway patency, prevent soft tissue collapse, and preserve the contour of reconstructed nasal passages. These appliances contribute significantly to both functional and esthetic rehabilitation [22].

Trismus stents or dynamic jaw-opening appliances are indicated for patients with restricted mouth opening resulting from trauma, surgery, oral submucous fibrosis, or radiation therapy. By delivering controlled intermittent stretching forces, these appliances improve mandibular mobility, facilitate physiotherapy, and enhance oral function [23].

Collectively, these specialized stents demonstrate the expanding scope of prosthodontics beyond conventional prosthetic rehabilitation, emphasizing the specialty’s integral role in multidisciplinary patient care.

Oral appliance therapy

Oral appliance therapy has become an established, conservative, and evidence-based treatment modality for the management of obstructive sleep apnea (OSA) and primary snoring. The prosthodontist plays a central role in patient assessment, appliance selection, fabrication, titration, and long-term follow-up, working in collaboration with sleep physicians and otolaryngologists. Current clinical practice guidelines recommend oral appliance therapy primarily for patients with mild to moderate OSA and individuals who are intolerant of continuous positive airway pressure (CPAP) therapy [11-13].

The most commonly prescribed oral appliances include mandibular advancement devices (MADs) and tongue-retaining devices (TRDs). MADs improve upper airway patency by advancing the mandible during sleep, thereby repositioning the tongue and associated soft tissues anteriorly while reducing pharyngeal airway collapse. Conversely, TRDs maintain the tongue in a forward position using negative pressure and are particularly useful in selected patients who are unable to tolerate MADs [24].

Several randomized clinical trials and systematic reviews have demonstrated that oral appliance therapy significantly reduces the apnea-hypopnea index (AHI), snoring intensity, and daytime somnolence and improves overall quality of life. Although CPAP remains the gold standard because of its superior efficacy in eliminating apneic events, oral appliances often demonstrate greater long-term patient adherence owing to their comfort, portability, and ease of use [11,16].

Successful treatment depends on appropriate patient selection, adequate mandibular protrusion, appliance titration, and periodic clinical review. Factors associated with favorable treatment outcomes include mild to moderate disease severity, a lower body mass index, positional OSA, and favorable craniofacial morphology [25].

Despite their clinical effectiveness, oral appliances are associated with certain adverse effects, including transient mandibular discomfort, hypersalivation, xerostomia, occlusal changes, and dental movement following prolonged use. Consequently, long-term follow-up is essential to monitor occlusal stability, assess patient compliance, and ensure sustained therapeutic success.

Diagnostic and provisional prosthodontic appliances

Diagnostic and provisional prosthodontic appliances are indispensable adjuncts in comprehensive prosthodontic rehabilitation, serving as reversible tools for evaluating treatment outcomes before definitive therapy. These appliances facilitate the assessment of occlusal relationships, vertical dimension of occlusion, centric relation, esthetics, phonetics, and neuromuscular adaptation, thereby minimizing the risk of irreversible treatment errors. They are particularly valuable in full-mouth rehabilitation, extensive fixed prosthodontics, implant-supported restorations, and patients requiring alteration of the vertical dimension of occlusion [26].

Diagnostic wax-ups and occlusal mock-ups provide a three-dimensional representation of the proposed prosthesis, allowing visualization of occlusal morphology, tooth position, and esthetic outcomes before tooth preparation. Similarly, provisional restorations and provisional splints enable clinicians to evaluate functional occlusion, anterior guidance, speech, masticatory efficiency, and patient adaptation under dynamic clinical conditions. This staged approach enhances treatment predictability while allowing necessary modifications before fabrication of the definitive prosthesis [18,19].

Recent advances in digital dentistry have further refined diagnostic procedures through the incorporation of digital smile design, virtual articulators, and CAD/CAM-based provisional restorations. These technologies facilitate the virtual simulation of treatment outcomes, improve clinician-technician communication, and enhance patient understanding and acceptance of proposed treatment plans [18,19].

Orthotic appliances for TMDs

Orthotic appliances constitute an important component of the conservative prosthodontic management of TMDs. Their principal objective is to provide reversible therapy by redistributing occlusal forces, reducing abnormal muscle activity, improving mandibular stability, and decreasing excessive loading of the temporomandibular joint. Unlike irreversible occlusal therapies, orthotic appliances permit continuous evaluation and modification according to the patient’s clinical response [18,19].

Mandibular orthopedic repositioning appliances (MORAs) are indicated in selected patients with internal derangement of the temporomandibular joint to establish a more favorable condylar position and reduce joint loading. Bite plane appliances are frequently prescribed for patients with deep bite, occlusal discrepancies, or parafunctional habits to disengage the posterior teeth, reduce elevator muscle hyperactivity, and promote neuromuscular relaxation [18,19].

Although orthotic appliances have demonstrated effectiveness in reducing pain and improving mandibular function, prolonged or inappropriate use may result in undesirable occlusal alterations, emphasizing the importance of accurate diagnosis, careful patient selection, and periodic clinical review. Current evidence supports their use as part of a comprehensive conservative management strategy rather than as definitive treatment [18,19].

Digital technologies in contemporary prosthodontic appliances

The rapid evolution of digital dentistry has transformed the design, fabrication, and clinical application of prosthodontic appliances. Contemporary digital workflows integrate CBCT, intraoral scanning, CAD/CAM, virtual articulation, digital occlusal analysis, and additive manufacturing (three-dimensional printing) to improve diagnostic accuracy, appliance fit, and treatment predictability [27,28].

Digital workflows generally begin with the acquisition of clinical records using intraoral scanners and CBCT imaging. The resulting STL and DICOM datasets are digitally integrated to generate a virtual three-dimensional representation of the patient’s dentition and supporting structures. This virtual environment enables prosthetically driven treatment planning, precise appliance design, and accurate fabrication while minimizing errors associated with conventional impression techniques [27,28].

CAD/CAM technology has significantly improved the fabrication of occlusal splints, surgical guides, radiotherapy stents, and provisional restorations by providing superior dimensional accuracy, standardized thickness, enhanced adaptation, and reduced chairside adjustment. Likewise, additive manufacturing technologies permit the rapid production of highly customized prosthodontic appliances with excellent reproducibility and efficient material utilization [27,28].

Digital occlusal analysis systems have further enhanced appliance therapy by providing an objective assessment of occlusal contact timing, force distribution, and functional occlusal relationships. These systems facilitate accurate adjustment of occlusal splints and complex prosthodontic restorations, thereby improving clinical precision and long-term treatment outcomes [29].

Artificial intelligence (AI) represents an emerging adjunct in digital prosthodontics. AI-assisted applications are increasingly being explored for automated landmark identification, virtual treatment planning, implant guide design, occlusal prediction, and appliance optimization. Although these technologies remain in the early stages of clinical implementation, they demonstrate considerable potential for improving diagnostic consistency, reducing operator-dependent variability, and facilitating individualized prosthodontic treatment planning [30].

Collectively, these digital innovations have shifted prosthodontic appliance therapy from conventional laboratory-based procedures toward a patient-specific, digitally integrated workflow characterized by improved precision, reproducibility, efficiency, and interdisciplinary communication [27-30].

Limitations and future directions

Despite substantial advances in appliance design and digital fabrication, several challenges continue to limit the widespread standardization of prosthodontic appliance therapy. Considerable variability exists in appliance design, material selection, fabrication protocols, treatment duration, and methods of clinical outcome assessment. Such heterogeneity limits comparisons between studies and complicates the development of universally accepted evidence-based clinical guidelines [27-30].

Although digital workflows offer significant advantages in precision and reproducibility, they are associated with increased equipment costs, software dependency, the need for technical expertise, and potential cumulative errors during data acquisition, virtual planning, manufacturing, and clinical transfer. Furthermore, long-term comparative evidence evaluating conventionally fabricated and digitally manufactured prosthodontic appliances remains limited [27-30].

Future investigations should focus on establishing standardized fabrication protocols, conducting multicenter randomized clinical trials with long-term follow-up, and developing uniform outcome assessment criteria for various prosthodontic appliances. The continued integration of artificial intelligence, advanced biomaterials, digital occlusal analysis, and additive manufacturing is expected to further enhance appliance precision, clinical effectiveness, and personalized patient care [27-30].

## Conclusions

Prosthodontic appliances, including splints, stents, and oral appliances, have evolved from simple supportive devices into indispensable components of contemporary prosthodontic practice. Their applications now extend beyond conventional rehabilitation to encompass the management of TMDs, bruxism, implant-guided surgery, maxillofacial oncology, obstructive sleep apnea, and complex restorative procedures. Current evidence supports their role in improving diagnostic accuracy, enhancing treatment predictability, protecting oral tissues, and facilitating functional rehabilitation through conservative and reversible therapeutic approaches.

The integration of digital technologies, including CBCT, CAD/CAM, intraoral scanning, additive manufacturing, digital occlusal analysis, and emerging artificial intelligence applications, has substantially enhanced the precision, reproducibility, and customization of prosthodontic appliances. These innovations have enabled prosthetically driven treatment planning, improved interdisciplinary collaboration, and expanded the scope of prosthodontic care toward patient-specific, evidence-based treatment protocols.

Despite these advances, challenges remain because of variability in appliance design, material selection, fabrication techniques, and clinical outcome measures. The absence of universally accepted standardized protocols and the limited availability of long-term comparative clinical evidence continue to hinder the establishment of definitive clinical guidelines.

Future research should prioritize well-designed multicenter randomized clinical trials, standardized fabrication and evaluation protocols, and continued investigation into advanced biomaterials, artificial intelligence-assisted planning, and digital manufacturing technologies. Such developments are expected to further strengthen the scientific foundation of prosthodontic appliance therapy and support its transition toward precision-based, digitally integrated, and patient-centered clinical practice.
